# Substrate requirements for let-7 function in the developing zebrafish embryo

**DOI:** 10.1093/nar/gkw173

**Published:** 2016-03-11

**Authors:** Wigard P. Kloosterman, Erno Wienholds, René F. Ketting, Ronald H.A. Plasterk

**Affiliations:** The Hubrecht Laboratory, Centre for Biomedical Genetics, 3584 CT Utrecht, The Netherlands

Nucl. Acids Res. (2004) 32 (21): 6284–6291. doi:10.1093/nar/gkh968

The Editors express a note of concern regarding the above article.

The Editors wish to alert the readers that the authors have raised questions about the validity of some of the figures presented in this article. Figures 3, 4, and 5b appear to contain duplicate images representing different experimental results, as well as additional signs of unacceptable splicing. As the laboratory that originally reported the work no longer exists, we are unable to request a more in-depth investigation of the original experiments. The Editors consider that this does not necessarily affect the validity of the results and the conclusions of the study, however we wish to alert our readership to these concerns.

Keith Fox, Senior Executive Editor, *Nucleic Acids Research*

Barry Stoddard, Senior Executive Editor, *Nucleic Acids Research*

